# A “twisted” microfluidic mixer suitable for a wide range of flow rate applications

**DOI:** 10.1063/1.4954812

**Published:** 2016-06-27

**Authors:** Shilpa Sivashankar, Sumeyra Agambayev, Yousof Mashraei, Er Qiang Li, Sigurdur T. Thoroddsen, Khaled Nabil Salama

**Affiliations:** 1 Computer, Electrical and Mathematical Science and Engineering Division (CEMSE), King Abdullah University of Science and Technology (KAUST), Thuwal, Saudi Arabia; 2 Physical Science and Engineering Division (PSE), King Abdullah University of Science and Technology (KAUST), Thuwal, Saudi Arabia

## Abstract

This paper proposes a new “twisted” 3D microfluidic mixer fabricated by a laser writing/microfabrication technique. Effective and efficient mixing using the twisted micromixers can be obtained by combining two general chaotic mixing mechanisms: splitting/recombining and chaotic advection. The lamination of mixer units provides the splitting and recombination mechanism when the quadrant of circles is arranged in a two-layered serial arrangement of mixing units. The overall 3D path of the microchannel introduces the advection. An experimental investigation using chemical solutions revealed that these novel 3D passive microfluidic mixers were stable and could be operated at a wide range of flow rates. This micromixer finds application in the manipulation of tiny volumes of liquids that are crucial in diagnostics. The mixing performance was evaluated by dye visualization, and using a pH test that determined the chemical reaction of the solutions. A comparison of the tornado-mixer with this twisted micromixer was made to evaluate the efficiency of mixing. The efficiency of mixing was calculated within the channel by acquiring intensities using ImageJ software. Results suggested that efficient mixing can be obtained when more than 3 units were consecutively placed. The geometry of the device, which has a length of 30 mm, enables the device to be integrated with micro total analysis systems and other lab-on-chip devices.

## I. INTRODUCTION

In recent years, microfluidics have made great advances in the field of biomedical diagnostic studies through the development of miniaturized microfluidic and nanofluidic biosensors.^1^ The channel dimensions in microfluidics enable the surface area to volume ratios to be significantly increased, thereby reducing the sample or reagent consumption and allowing the development of compact and portable devices. However, in such small channels, the majority of flow occurring is laminar, due to small Reynolds (Re) numbers. This is in contrast to the traditional turbulent flow required for the efficient mixing of fluids. Therefore, introducing vortices at moderate Re numbers within the channel would form an approach to providing efficient mixing. Mixing at a fast and controllable rate is currently essential for the development of microfluidic and lab-on-chip devices often used in assays involving many reagents and samples. Microfluidic devices will significantly influence the future of the biological process industry.^2^ Amongst these, microfluidic mixers which allow the rapid mixing of fluids within the microchannel find application in many biochemical processes, including the synthesis or sequencing of nucleic acids,^3^ chemical reactions,^4^ DNA purification,^5^ and polymerase chain reaction.^6^ A further advantage of integrating microfluidic mixers is a reduction in the incubation time for bead-based enzyme-linked immunosorbent assays (ELISA) used in the study of cancer biomarkers.^7^

In order to efficiently mix reagents and samples, many active mixers have been designed and tested, such as ultrasonic,^8^ magnetic stirring,^9^ and bubble-induced acoustic actuation.^10^ However, compared to active mixers, passive mixers are preferred due to their low-cost, ease of fabrication, and integration within microfluidics. They require no complex control units or additional power input. The majority of the 3D passive mixers^4,11–14^ utilize multiple splitting, recombining, and rotating channels for the efficient mixing of fluids. Hence, these micromixers show an acceptable mixing performance only at slow flow rates and on the microscale, while the ideal micromixer should be independent of variations in flow rate. Efforts have been made to develop 2D passive micromixers using radial baffles.^15^ Fabrication of 2D micromixers with grooves^16–19^ on the channel surface introduces chaotic flow within the channel. These micromixers are not compatible with low flow rates, as they cannot form vortices, and the contact surface between the streams is usually linear, without rotation or expansion of fluids within the channel. However, these micromixers can operate at high flow rates. Therefore, the flow induced within the channel by frequently rotating, splitting, and recombining the fluids allows the mixers to operate at a wide range of flow rates. Although a number of these micromixers have been built with two or three layers of polydimethylsiloxane (PDMS) or grooves, and are quite small, these are only applicable to high flow rates, which^20,21^ favor the transition of the flow within the channels. Micromixer applications are not limited to chemical reactors,^22^ but are also helpful in the performance of enzyme reactions,^23^ biological analyses,^24,25^ and drug delivery,^26^ and these applications do not always require fixed flow rates during the process. The demand has therefore arisen for a micromixer that can operate at different flow rates, which are required for a broad range of applications.

Fabrication of a microfluidic channel is an important aspect of developing a microfluidic device. Many fabricating techniques have been adopted in recent years, which include time-consuming techniques such as photolithography,^27^ etching,^28^ and the computer numerical control (CNC) micro-milling method.^29^ Laser-directed writing is a good tool choice for micromachining, as it provides a simple and inexpensive method of operation.^30^ Ultrafast femtosecond laser processing,^31^ as well as IR lasers and UV lasers, have been used to fabricate devices based on their applications.^32–34^ Direct laser writing has several advantages over the lithographical process. Lithography limits the designs of the microchannel to single-depth planar geometries. The uniform depth of the microchannels results in non-physiological flow patterns. It is also possible to build multistep microchannels using lithographic techniques; however, the masks used are expensive, and the process is tedious. The channels can also be fabricated using embossing and imprinting techniques^35^ or the injection-molding technique,^36^ which are also tedious and time-consuming. To overcome the above limitations, the Universal Laser System was used in the current study to write directly onto the PMMA, forming microchannels. These were then thermally bonded in order to obtain micromixers. Laser penetration depth can control the depth of the microchannels. These interconnected pervasive 3D micromixers may find widespread application in microfluidic devices. The design developed in this study is that of a modified tornado mixer,^37^ which is termed the “twisted” mixer. The limitations involved in using fewer units and the effects of this on mixing and on the flow rate will be discussed below.

This paper describes a 3D micromixer based on the principle of *splitting/recombination* and *chaotic advection.* The contours of the gradient at different sections are used to enumerate the diffusing trend of the solute. ImageJ is used to illustrate where the diffusion occurs. The number of units that provides acceptable mixing within the described micromixers is evaluated via simulation. The experimental results and simulations confirm that the micromixers are suitable to be used for a wide range of flow rates and various viscosity fluids. The micromixers are fabricated using the direct laser writing technique; this was therefore rapid. The overall length of the channel is 30 mm, which simplifies the integration of this device with other devices. Experiments are performed in order to evaluate the performance of mixing quantitatively under real conditions. The results show that the mixer can operate at a wide range of flow rates.

## II. EXPERIMENTAL METHODS

### A. Sample sites

Commercial food dyes were used. Citric acid and sodium hydroxide pellets were purchased from Sigma, and phenolphthalein from ACROS Organics. All the solutions were prepared by diluting with deionized (DI) water.

### B. Preparation of solutions

Phenolphthalein in its native form was obtained in the powdered form. A 1% solution of phenolphthalein was used as the indicator. To achieve this concentration, 1.0 g of phenolphthalein was dissolved in a 50% ethanolic solution and a prepared 1% indicator solution, and was stored in an eyedropper bottle. Citric acid (0.05 M) was prepared and added to 9.90 ml of citric acid solution; 10 *μ*l of prepared 1% phenolphthalein was added as an indicator. NaOH 1 M solution was used for the basic solution in the pH test experiment. Glycerol (98% v/v) was mixed with green food grade dye to demonstrate mixing with water.

### C. Fabrication

**FIG. 1.**
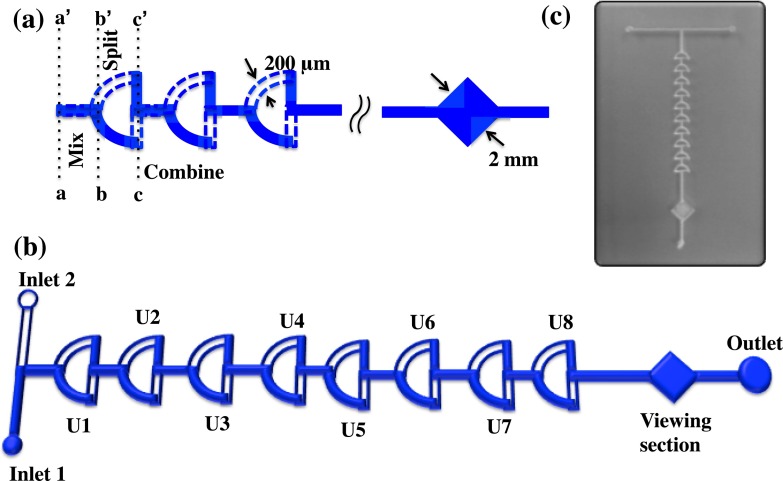
(a) Illustration of the microfluidic mixer showing different regions. Shaded area is the bottom layer of the mixer and the dotted line represents the top layer of the mixer. (b) Mixer with ambient mixing units. (c) Prototype of the fabricated mixer.

**FIG. 2.**
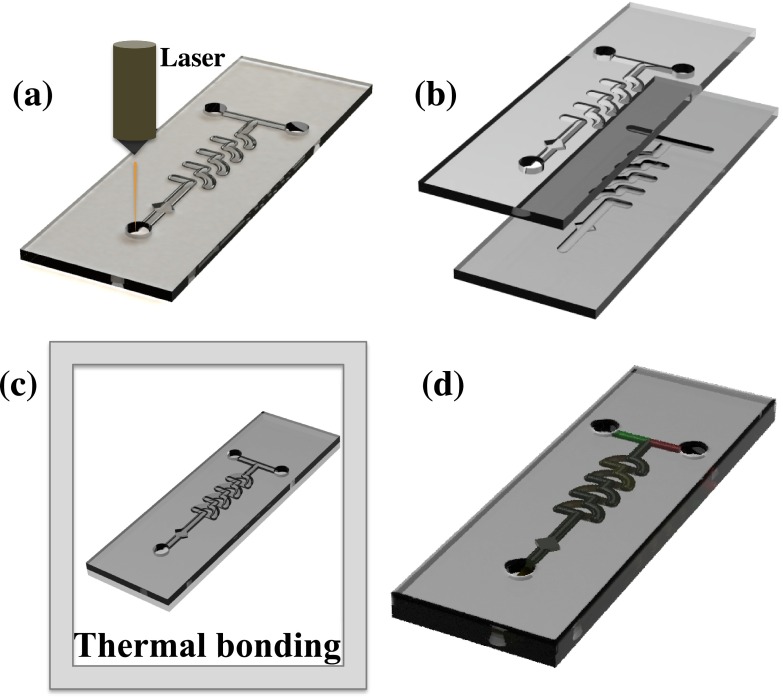
(a) Laser writing of base layer. (b) Drills and channels etched using laser. (c) Thermal bonding of base and top layer. (d) Illustration of the fabricated chip.

The Universal CO_2_ laser system was used to cut the channels into the PMMA sheets, which had a thickness of 2.0 mm. CorelDraw software was used to design the channels. The micromixer was composed of successively arranged “quadrant of a circle” shape mixing units in two layers. The design file was opened in the UCP (Universal Laser Systems Control Panel) program to laser write the channels onto the PMMA. A 60% power, 40% speed, and 1.0 mm z-axis were set to obtain 200 *μ*m-deep and 200 *μ*m-wide channels. The overall microchannel path of the arranged mixing units formed a 3D path, and the micromixer consisted of combining, splitting, rotating, and recombining regions, as shown in Fig. 1(a). This was designated as one unit (U1); each unit was 1.3 mm in height. The dimensions of the channel are shown in Fig. 1(b). After the laser writing process was completed, the PMMA base and top layer were thermally bonded together. The parameters for alignment, bonding, and tubing will be discussed below. The volume required to fill the mixer was small (approximately 20 *μ*l) and could therefore be used for diagnostic applications. The geometry of the proposed mixers has the ability to mix larger non-soluble particles such as cells. This is an important consideration for the study of the dynamic and complex interplay among cell populations.^38^ A prototype of the fabricated chip is shown in Fig. 1(c). The detailed fabrication procedure is illustrated in Fig. 2.

The two PMMA layers were adhered using chloroform glue.^39^ Small volumes (5.0 *μ*l) of chloroform were dropped into the corners of the device between the top and bottom layers and aligned using an optical microscope, before evaporation of the chloroform, i.e., within 30 s. Adding more than 10 *μ*l chloroform or adding chloroform near the channels resulted in clogging of the channels. The devices were then left unmoved for approximately 1 min to allow them to attach. This chloroform adhesion allowed the precise alignment of the channels and also prevented the PMMA layers from becoming dislocated while under pressure. The glued PMMA devices were sandwiched between glass plates in order to prevent adhesion of the device to the heating plates, and placed exactly at the center of the press-stage. The temperature was gradually raised to 150 °C within approximately 45 min under 59 lbs of pressure. At this temperature, the PMMA melted slightly, and the substrate was in a near-molten state. The pressure was then increased to 151 lbs for 20 s to allow the molten PMMA interface layers to attached to each other. The pressure was then immediately lowered to zero for approximately 5.0 s. The pressure was then increased to 50 lbs. The substrate was cooled slowly to avoid bending of the substrate and to prevent bubble formation within the microfluidic chip. This thermal bonding method between the PMMA substrates provided adequate, strong bonding, reducing the likelihood of it detaching. Furthermore, it prevented leakage between the stacked layers. These conditions were obtained after many optimization trials.

Inlet and outlet holes of 0.9 mm in diameter were drilled using the laser on the top layer of the device. A stainless steel blunt needle with a 1.0 mm outer diameter was then force-fixed to the inlet/outlet, after bonding the device. The blunt needles and Tygon tubes (with a 1.0 mm inner diameter and a 2.0 mm outer diameter) were adhered using superglue. The thermally bonded device and the blunt needle were again fixed using superglue; this prevented any possible leakage in the device.

### D. Simulation

**FIG. 3.**
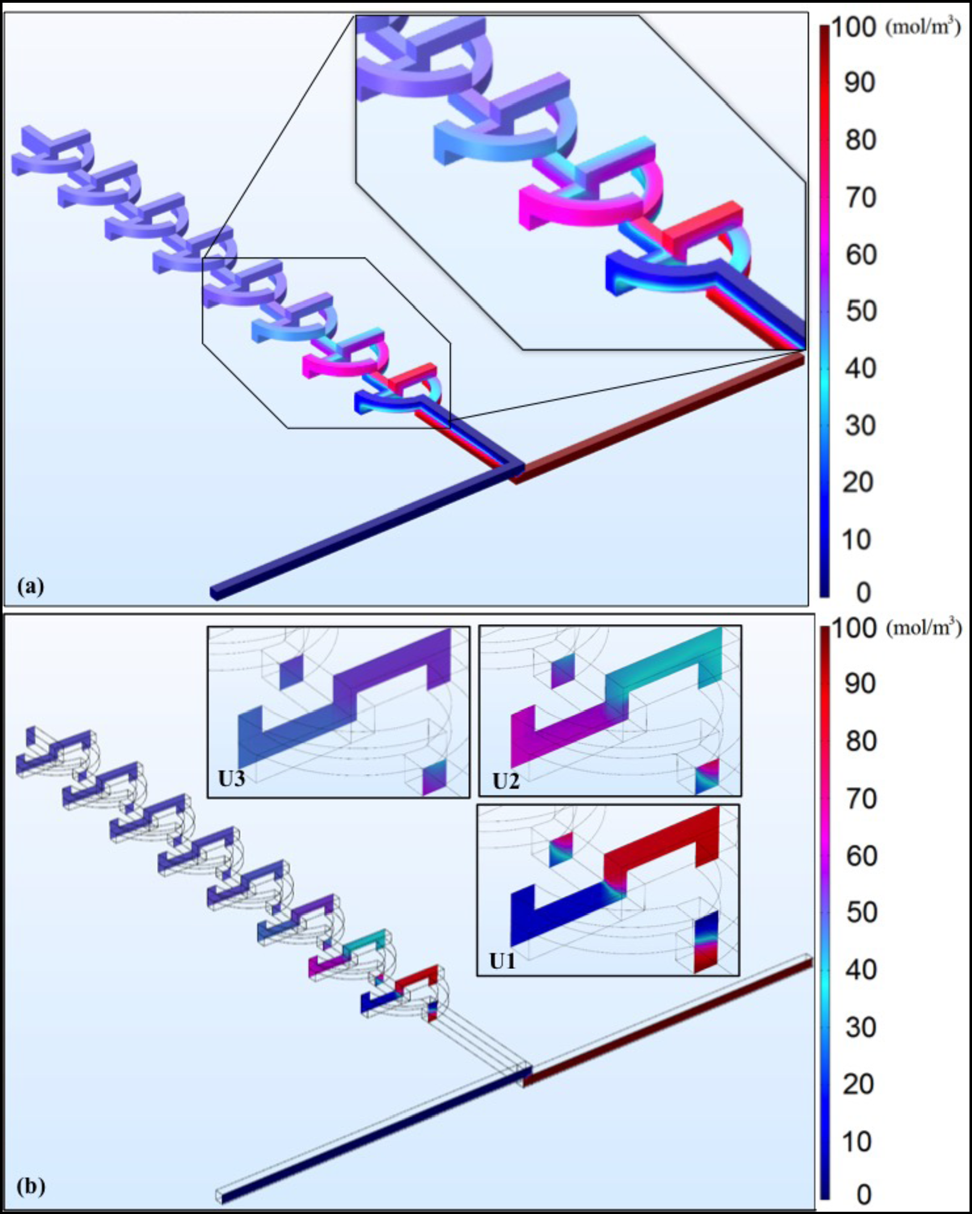
Simulation results (a) design and concentration profile of the 3D mixer, with an enlarged view of the first 3 units showing the concentration profiles varying in the units (b) flow concentration profiles at cross sections for the first 3 units.

Simulations were performed with the COMSOL Multiphysics commercial software (COMSOL 5.2) in order to quantify the mixing performance of the micromixer. The mixing profile within the unit was investigated using 3D models, as depicted in Fig. 1. In the numerical simulation models, the type of the fluid used was an incompressible Newton fluid governed by the Navier-Stokes equation.^40^ The component of the fluid is water with a kinetic viscosity of v = 1 × 10^−6^ m^2^/s at room temperature. The concentrations of the two different fluids to be mixed were set as C = 0 mol/m^3^ and C = 100 mol/m^3^ at inlet 1 and inlet 2, respectively, while the diffusion coefficient of the solute in water was D = 2.3 × 10^−9^ m^2^/s.^41^ The channel design matched the fabricated mixer, as shown in Fig. 1. The mixer had a fixed input area of 200 *μ*m × 200 *μ*m. The units were 600 *μ*m apart, and the arc radius of the mixer was 1000 *μ*m, with an effective channel length of ∼785 *μ*m. The concentration of the input specimen was 100 mol/m^3^, and the liquid medium was water. There were two inputs, which would sustain a laminar inflow, with a volumetric flow rate of 100 *μ*l/min, and one outlet. The result of mixing different concentrations is shown in Fig. 3(a). It was evident that three mixer units were necessary to achieve full mixing. In the inset of Fig. 3(a), the first three units of the mixer are enlarged to show the concentration flow profile within the mixer. After the third unit, the flow concentration was almost unchanged throughout the mixer. Fig. 3(b) further shows the flow concentration profile after each unit (U1, U2, and U3), revealing the remarkable mixing efficiency within the first three units.

### E. Experimental calculations

The equations were formulated with reference to previously published articles.^42,43^ The histogram matrix was retrieved using the ImageJ software, with the pixel intensity weight for the particular color intensity. Pixel intensity was then calculated using Equation (1), below. The intensity obtained from ImageJ was incorporated into Equation (2) to calculate the mixing efficiency. The progress of the mixing efficiency with the number of mixer units was then determined.

An optical method was used for testing the efficiency of the mixer. The mixing pictures were recorded using a digital camera (Olympus Stylus) that captured 3D devices in a single focused image by stacking the images layer upon layer. The mixing efficiency was measured by

$$\sigma =1-\sqrt{\frac{1}{N}\sum_{i=1}^{N}{\left(\sigma_{i}\right)}^{2}},$$ (1)

where $\sigma_{i}$ is the total deviation at pixel i

$$\sigma_{i}=\frac{\left(I_{i}-I_{mix}\right)}{\left(I_{unmix}-I_{mix}\right)},$$ (2)

where $I_{i}$ is the pixel intensity of the mixing picture, $I_{unmix}$ is the intensity prior to mixing, and $I_{mix}$ is the intensity after complete mixing.

In order to calculate $\sigma_{i,}$ the intensity of the images at pixel i was obtained via histogram for an area of 250 pixels using the ImageJ software. $I_{unmix}$ referred to the intensity at the unmixed arm of the mixer (in this case, the left arm was used, as it was positioned at the bottom layer and hence was uniform with the remaining units to be calculated). $I_{mix}$ was the intensity at each unit after mixing.

## III. RESULTS AND DISCUSSION

Although the device fabrication was simple, a number of parameters needed to be considered during the fabrication of these devices. Laser fabrication is dependent on four factors: (1) the focal plane of the substrate, (2) power, (3) the pulse rate, and (4) the writing speed on a substrate. In the current study, a 10.6 *μ*m CO_2_ laser that generated a maximum output of 70 W at 1000 pulses/s was utilized. The parameters were optimized to 60% power, 40% speed, and a 1.0 mm-thick substrate, after trial and error. This was found to be highly replicable when all parameters were optimized. In this case, a highly reproducible device with a depth and width of 200 *μ*m was achieved. Many devices can be simultaneously laser etched. Approximately 15 min was required to laser etch a complete set of up to 30 devices in the same run. The thermal bonding process could be performed on approximately eight devices in parallel, requiring approximately 1.5 h. Multiple whole devices (up to eight) could be printed and assembled within 2 h.

**FIG. 4.**
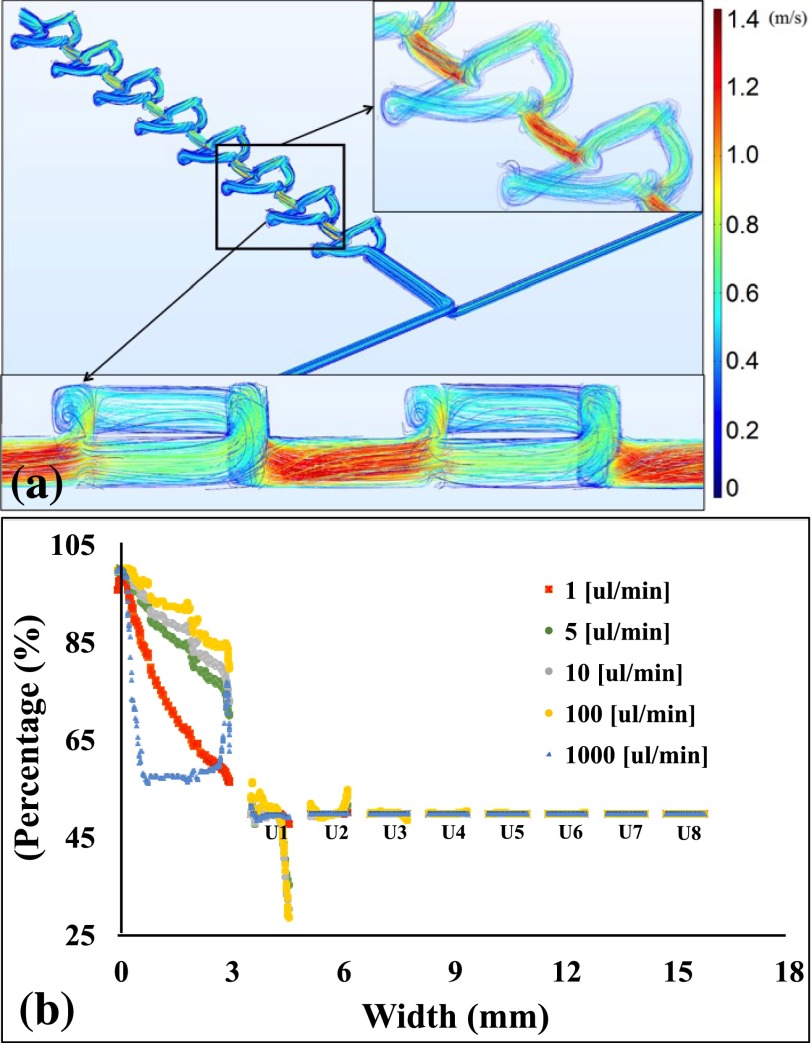
(a) Simulation results showing vortices at the edges of the mixer unit. (b) Simulation results revealing the concentration profile after each mixer unit of mixer.

The mixing efficiency in the twisted mixer was enhanced due to the vortices generated at the edge of the arc-shaped channels, where splitting/recombining induces a chaotic advection in the region. Simulation results for the generated vortices are shown in Fig. 4(a). These simulation results did not account for the surface roughness formed by the nature of the fabrication process; however, it was observed that the surface roughness could also induce local eddies and vortices.^44^ Variable flow rates were evaluated using the concentration values along a centerline drawn through the mixer units. The mixing concentrations are plotted in Fig. 4(b).

We simulated (Fig. 4(b)) different flow rates in the device. The results agreed with the experimental results, except for those at 1.0 ml/min. The simulation showed that mixing occurs in the first unit, while the experimental results revealed a much lower mixing efficiency at 1.0 ml/min. This deviation in the experimental results may have been due to the ideal conditions used during simulation. This mixer appeared to show good mixing efficiency through enhanced diffusion and rotation of fluids at the edges of the arc-shaped channels. Furthermore, as this mixer provided an approximately 75% mixing efficiency at 1.0 ml/min, it is also suitable for high flow rate applications.

To verify and observe the mixing efficiency of the proposed mixers, two tests were performed: (1) a dye visualization test and (2) a pH test. The mixing efficiency was also observed to vary with the number of units, the dimension of the channels, and the height of the units. All the parameters mentioned above will now be further discussed. From the design of the micromixer, as shown in Fig. 1, the flow profile of the specimen within the channels could be visualized. At the inlets, the specimen had a linear flow for a distance of approximately 0.5 mm; the two fluids then converged into one channel, causing an initial mixing between the region bordered by a,a′ and b,b′. Furthermore, the fluids split into two directions, with half of the fluid volume moving through the top layer and half through the bottom layer of the mixer between the region bordered by b,b′ and c,c′. The fluid in this region already contained an amount of specimen exchanged from the previous region. At c,c′, the fluids recombined and repeated the cycle with the number of units present in the mixer. This guaranteed the occurrence of chaotic advection in the mixer. The mixing was based on the number of units arranged in a linear pattern, different flow rates and on the injection of highly viscous fluids; this will be discussed further below. The proposed mixers can also find application in monitoring diffusion processes and mixing behaviors within the microfluidic channel in a dye-free environment.^45^

### A. Variation in number of units

**FIG. 5.**
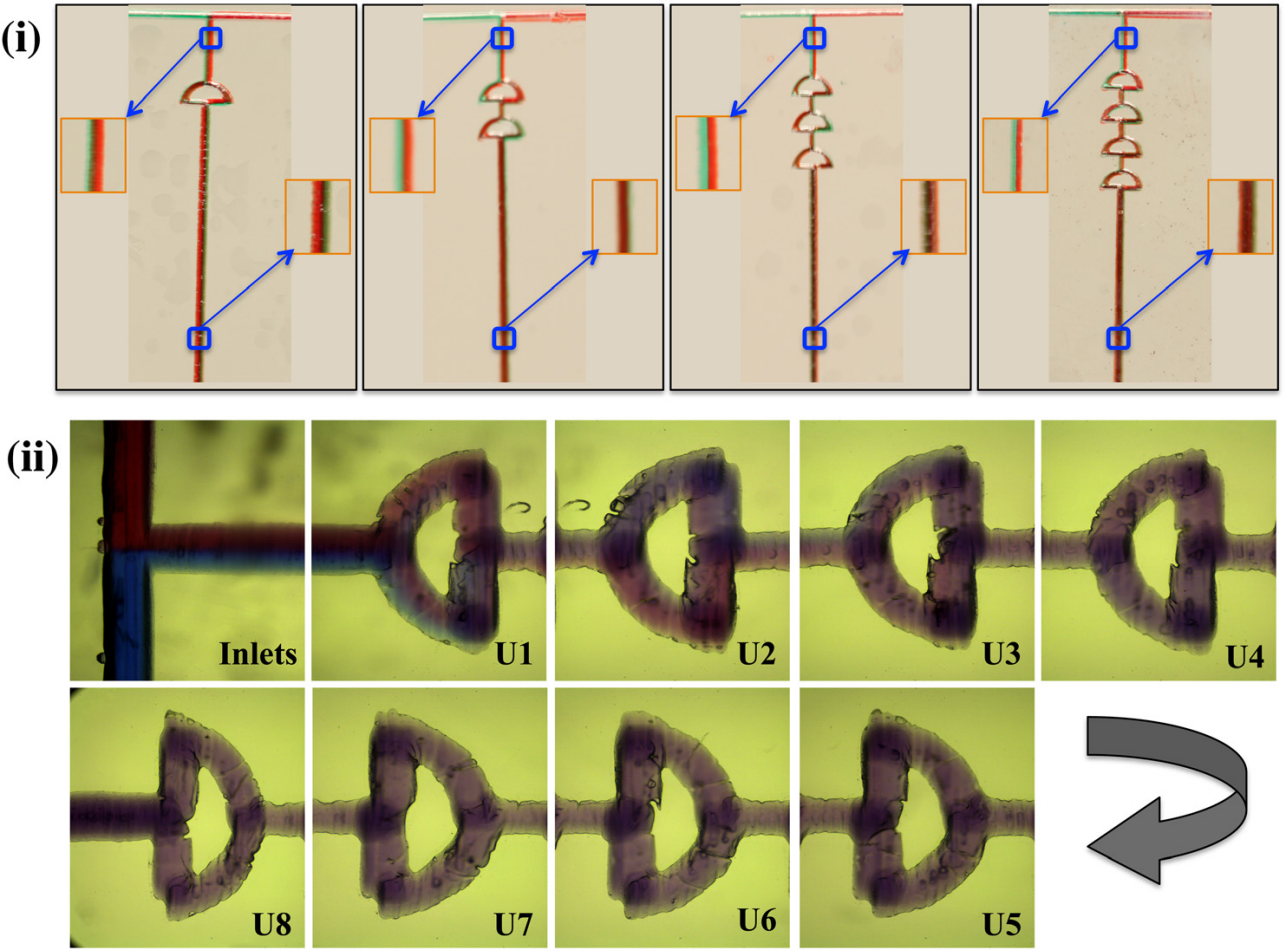
(i) Mixing efficiency of fluids in different units (a), (b), (c), and (d) representing 1, 2, 3, and 4 units, respectively (an enlarged view of the selected section is shown to allow the observation that the fluid flow in the separate streams in the neck region and thereby combined to form a single color with the addition of mixer units). (ii) Images showing mixing within channels captured via a high-speed color video camera (Photron SA3).

The mixing efficiency varied with the number of units arranged in series. When the three-unit mixer was used, an efficient mixing phenomenon was observed, and was then confirmed via simulation. The mixing phenomenon in one, two, three, and four-unit mixers are shown in Fig. 5(i). The mixing phenomenon was unchanged when more than six units were used. To ensure proper mixing, an eight-unit mixer was used for all experiments. The mixing of fluids was captured via a high-speed color video camera (Photron SA3) with a frame rate of 2000 frames per second (Fig. 5).^22^ The camera was equipped with a long-distance microscope using a Mitutoyo 5× objective, giving a corresponding pixel resolution of 3.5 *μ*m. Various devices fabricated with the optimized parameters of the laser and thermal bonding are shown in Fig. 5. The fabrication process was highly replicable, and the mixing efficiency was almost identical in the three trials performed.

The neck region, prior to entering the first unit, is a 3D configuration. An enlarged image of the section prior to entering the first unit and after leaving their respective units is shown (Fig. 5(i-a–d)). The dyes were distinguishable in all images prior to their entering the first unit (Fig. 5(i-a–d)), whereas the mixing of fluids after leaving the unit differed between units. In Fig. 5(i-a), the different unmixed streams can be seen after they pass through the first unit. The fluids were mixed at the end of four units. In Fig. 5(ii), the inlets were fed with Sudan blue and Sudan red dyes. Again, the fluids flowed in separate streams before the first unit and started to combine after the first unit. Here, the mixing of the dyes is observed, as a slight amount of blue dye can be seen in the top arc of Fig. 5(ii-U2). The dyes were completely mixed, and the color seemed to remain unchanged after the third unit, which was evident through simulation and experimental results.

From the simulation results, it was obvious that the mixing of fluids was not highly efficient when using fewer than three units. The mixing efficiency was sub-optimal when the length of the units was approximately 1.0 mm. The liquids traveled short distances and did not have sufficient laminating area; hence, we opted to maintain the length of the arc-shaped mixers at 1.3 mm.

**FIG. 6.**
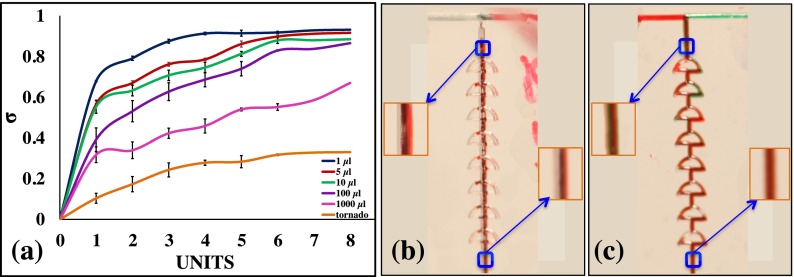
Mixing efficiency at different flow rates. (a) The flow of fluid in the tornado mixer at 1.0 ml/min. (b) The flow of fluid in the twisted mixer at 1.0 ml/min. Enlarged images of the selected sections are representative of the fluid flow within the channel.

These micromixers can operate at low (1.0 *μ*l/min) and high (1.0 ml/min) flow rates without disturbances in the mixing phenomenon. The flow quality is not negatively affected by the increasing flow rate, which would usually have an effect on lower mixing efficiency in the high flow rates.^20^ In order to demonstrate that the micromixers can operate with a wide range of flow rates, from 1.0 *μ*l/min to 100.0 *μ*l/min, a dye test was performed, and mixing was quantified using the above calculations. The mean grayscale values of the selected region were retrieved using ImageJ software. The mixing efficiency increased with the increase in units, which were arranged in a consecutive manner up to six units. After passing through the six units, the mixing phenomenon seemed to stabilize. The mixing of fluids in each unit at different flow rates is depicted in Fig. 6(a). The flow in the mixers was stable even at the high flow rates, while in some micromixers the mixing efficiency tended to be reduced in stability with the increasing flow rate.^21^ Steady flows were observed for flow rates from 1.0 to 1000.0 *μ*l/min. The efficiency of mixing with the tornado mixer was also compared; the flow of fluids within the mixer is represented in Fig. 6(b). The additional channel within the mixer units provided lower resistance to the fluids passing through the mixer, and could therefore have been the reason for the low mixing efficiency. The fluid completely filled the units and the change in intensity of the color at 1.0 ml/min is shown in Fig. 6(c). At the higher flow rates, the molecules did not have sufficient time to mix through molecular diffusion; hence, the efficiency of mixing was greatly reduced compared to the low flow rates. In the present mixer, at 1.0 *μ*l/min, the efficiency of the mixer was 0.80 after the first unit, as the diffusion of molecules had occurred and the fluids had traveled a relatively long distance to obtain that efficiency. It was also seen that the mixing efficiency of the tornado mixer increased slowly. However, despite the 3D configuration of the tornado chip, only its arcs are single layered, whereas the center-connecting channels are in two layers. It was here observed that the fluids tended to flow separately in the top and bottom layers, rather than swiftly up-and-down as observed in the twisted channel. This caused an error in the color intensity measurement, possibly due to the camera being unable to accurately obtain the true color intensity near the mid-plane region, where the majority of diffusion occurred. Consequently, the mixing efficiency of the tornado chip was expected to be undervalued; however, it remained lower than the efficiency of the twisted mixer. In examining the twisted mixer, it was obvious that with the addition of mixing units increased the mixing efficiency; after approximately five units, the mixing efficiency stabilized, indicating the fluids were well mixed (50% of each inlet), as observed in the simulation results. The splitting/recombining configuration of the microchannel generated vortices at moderate Re numbers; this may have been the reason for the high mixing efficiency of the twisted micromixers. A mixer that can operate at a wide range of flow rates could be integrated into a variety of applications that require high flow rates, such as microfluidic filtration,^46^ and applications that require low flow rates, such as cell cultures or vasculature on a chip.^47^ Therefore, this mixer is a possible device for the above applications.

### B. pH test

**FIG. 7.**
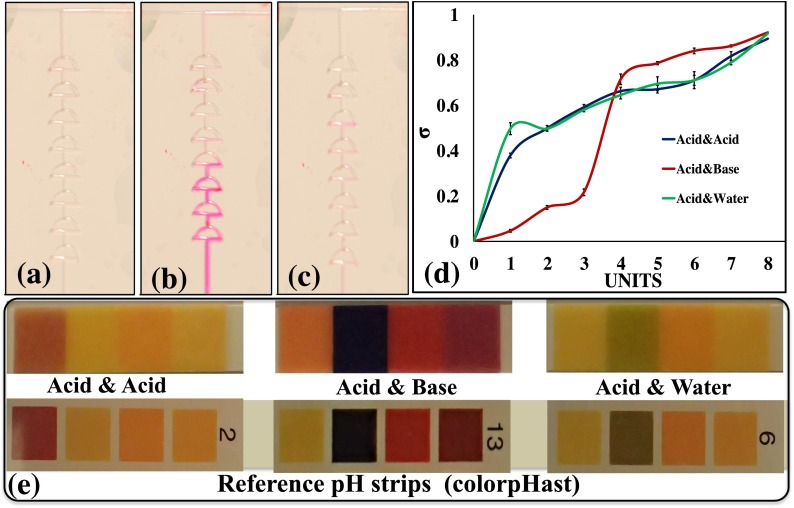
(a) Mixing of acid and acid; (b) mixing of acid and base; (c) mixing of acid and water; (d) plots of gray values of mixing in each case; and (e) change in pH compared with commercially available pH strips.

In order to evaluate the performance of the micromixers, pH tests were performed in which three different cases were demonstrated. The pH tests were conducted using phenolphthalein as an indicator.^10,21,48^ In each case, the color of the solution turned a dark pink with a basic solution, a light pink with a neutral solution, and remained colorless with an acidic solution. In case (1), the acidic solution (citric acid) with added phenolphthalein was introduced at inlet 1, and the basic solution (1 M NaOH) was introduced at inlet 2. Fusion 100 Touch Syringe Pumps were utilized throughout the experiment for the injection of fluids into the channel. In this case, the liquids turned a dark pink during mixing, based on the molarity of the pH used in inlet 2. In case (2), the acidic solution with added phenolphthalein was introduced at inlet 1, and DI water was introduced at inlet 2. In this case, the color of the solutions turned a light pink when mixed due to the neutral pH. In case (3), the acidic solution with added phenolphthalein was introduced at inlet 1 and another acidic solution (HCl) was introduced at inlet 2. The mixed solution remained colorless, as phenolphthalein does not change color in acidic solutions. The pH of the solution was also tested at the outlet using ColorpHast pH strips. The change in color in the devices for these three cases is represented in Figs. 7(a)–7(c). The mixing efficiency of the device was calculated using the above equations and plotted on a graph (Fig. 7(d)) to show the color variation in each mixing unit. Various pH solutions were used to test the reproducibility of the mixers. The solution was pre-prepared to a pH of 14, 7, and 1. The experiment was performed at the 5.0 *μ*l/min flow rate and utilized the optimized eight-unit mixer for further experimentation. The change in color of the pH strips and the reference color are shown in Fig. 7(e). In Fig. 7(e), the pH of the solution was observed to have changed due to the mixing, and the pH reference strips showed a pH of 13, 6, and 1, respectively.

The mixing of fluids, in this case, was based on three factors: (1) the reaction time, (2) the concentration of the reacting chemicals, and (3) the flow rate. When the flow rate was high (approximately 1.0 ml/min), the color was observed in the collection tube after it passed through the outlet. Therefore, all the figures were captured at 5.0 *μ*l/min. This provided sufficient time for the samples to react and express color after the reaction. A slight increasing trend was observed in the acid and base curve. However, the reaction was not observable with the naked eye, as the color in the acid and base solutions changed with the number of units. The sudden changes in color observed are presented in Fig. 7(b). In Fig. 7(c), a gradual increase in color is seen, because a strong acid reacts with water (a neutral solution) and the mixing between them yields a light pink. This light pink was again seen after three units of mixing; the curve shows the color changing from colorless to light pink.

### C. Viscous fluids

**FIG. 8.**
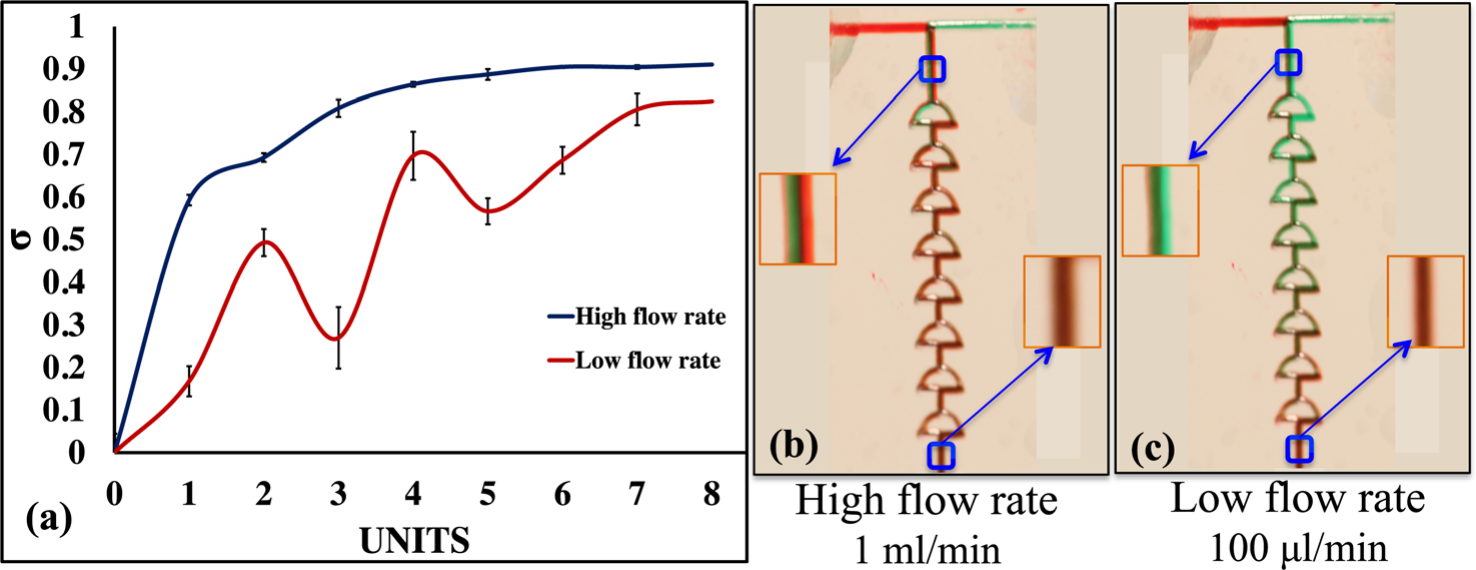
(a) Plots of gray values of mixing in each unit; (b) mixing of glycerol pre-mixed with green dye and water pre-mixed with red dye at 1.0 ml/min; and (c) mixing of glycerol pre-mixed with green dye and water pre-mixed with red dye at 100.0 *μ*l/min.

The mixing of various viscous fluids, i.e., glycerol 98% (v/v; viscosity of 919 cP) with water (viscosity of 1 cP) was demonstrated. The mixing of glycerol at high flow rates led to stable mixing efficiencies, whereas the mixing efficiency was unstable in low flow rates (first four units of the mixer). This mixer can therefore be used to mix highly viscous solutions when the fluids are allowed to mix at high flow rates (1.0 ml/min). The plot shows 50% mixing when the fluids were introduced at the high flow rate. The fluids started mixing before entering the first unit, and the efficiency reached approximately 60% after the first unit. The mixing efficiencies at 1000 *μ*l/min and 100 *μ*l/min were calculated after each unit; these are plotted in Fig. 8(a). Figs. 8(b) and 8(c) represent the mixing efficiencies at a high flow rate and low flow rate, respectively. The calculated results show that the mixing efficiency was unstable for lower viscous fluids introduced at low flow rates, and provided a mixing efficiency of >70% at the end of eight units. To further ensure stability, a mixer with >10 units can be used at low flow rates for highly viscous fluids. However, it should be noted that this color intensity detection method may not be accurate for highly viscous fluids. A pure green color can be seen on the left side of the first unit in Fig. 8(b). Furthermore, Fig. 8(a) shows the results of the low flow rate, indicating that the mixing efficiency fluctuated, which may not be physically possible. The detection methods may well resolve the color intensity at the top layer of the fluid, but may face limitations in accurately determining the fluid concentration profile underneath the top layer, especially for deeper channels. To allow an improved mixing of highly viscous fluids, the presence of a larger compartment located after the mixing channels would be preferred.^21^ While increasing the length of the channel in this case, by increasing the number of mixer units would be a further approach to increase the diffusivity of the solutes in fluids of different viscosities.

The proposed mixer provides appropriate circulation flow within the culture region in a peristaltic manner,^49^ which is critical for long-term cell growth. In addition, it provides a platform for the majority of biochemical analyses that involve liquid handling, i.e., reagents mixing in bioprocesses and bioengineering,^50^ long-term cell cultivation, and studies in which cells grow and react with introduced drugs for drug screening/delivery applications.^51^ As this mixer can be used to mix cells, it can be integrated into the continuous sensing of acute toxins in drinking water, and to mix cell suspensions and water samples.^52^

## IV. CONCLUSIONS

In conclusion, a mixer that operates at various flow rates and viscosities has been demonstrated. The time required to fabricate these devices is less than that required in photolithography techniques. The depth and width of the channels are approximately equal to those obtained using a photolithography method. Depending on the thickness of the substrate, the laser power and speed can be adjusted to achieve channels that can vary in dimension from 50 *μ*m to 300 *μ*m. Simulation results revealed that mixers with more than three units provide good mixing efficiency. Although many passive mixers have previously been developed, they did not exhibit adequate mixing efficiency at low flow rates, as the channel geometries did not result in visible chaotic advection. The streams usually cause the expansion and rotation of fluids with the surfaces rather than the vertical contact of fluids within the microchannels. Therefore, a mixer with appropriate geometry would be useful to enhance mixing efficiency at various flow rates and could be applied to on-chip tissue culture,^53^ and be integrated into micro-total analysis systems and lab-on-chip devices that involve the study of reaction kinetics, sample dilution, and improved reaction selectivity.
